# Machine learning and process-based modeling of spatiotemporal changes in active layer thickness across Alaska

**DOI:** 10.1038/s41598-025-26586-w

**Published:** 2025-11-27

**Authors:** Sagar Gautam, Umakant Mishra, Sarah N. Scott, Mark J. Lara

**Affiliations:** 1 Bioscience Division, Sandia National Laboratory, Livermore, CA 94550 USA; 2 Thermal/Fluids Science & Engineering, Sandia National Laboratory, Livermore, CA 94550 USA; 3 Department of Plant Biology, University of Illinois Urbana-Champaign, Urbana, IL 61801 USA; 4 Department of Geography, University of Illinois Urbana-Champaign, Urbana, 61801 United States

**Keywords:** Climate sciences, Ecology, Ecology, Environmental sciences

## Abstract

Permafrost degradation poses a growing threat to infrastructure stability and ecosystem resilience in the rapidly warming Arctic. We investigated the spatiotemporal dynamics of active layer thickness (ALT) across Alaska by integrating field observations, environmental datasets, a physically based Stefan model, and machine learning (ML) techniques. Using weather projections from the Coupled Model Intercomparison Project Phase 6 under two Shared Socioeconomic Pathways (SSP 2-4.5 and SSP 5-8.5), we assessed ALT sensitivity to projected future weather conditions. The random forest (RF) model outperformed the Stefan approach in predicting ALT on the training dataset (R² = 0.84 vs. 0.53) but demonstrated lower generalizability on the test dataset (R² = 0.24 vs. 0.54). The root mean square error (RMSE) for the RF model for training and testing ranged from 14 to 22 cm, compared to 17 and 18 cm for the Stefan model. Variable importance analysis revealed that mean annual temperature and slope angle were the strongest predictors of ALT, accounting for 19% and 18% of the variance, respectively, followed by sediment transport index (14%) and stream power index (11%). Comparative analysis of baseline ALT predictions showed the Stefan model tended to project a thicker active layer (mean ± SD: 65 ± 16 cm), compared to the RF model (mean ± SD: 59 ± 8.8) cm). Both models indicated a latitudinal gradient in ALT, with shallower depths at higher latitudes. Projected ALT increases by 2100 were estimated at 3.3 ± 2.2 cm under SSP 2-4.5 and 5.9 ± 4.0 cm under SSP 5-8.5 for the ML model, whereas the Stefan model projected substantially larger increases of 13 ± 2.6 cm (SSP 2-4.5) and 28 ± 4.4 cm (SSP5-8.5). Spatial analysis showed the greatest ALT increases in northern Alaska, with relatively smaller changes in southern regions. These findings highlight the complex, multifactorial nature of ALT dynamics and the value of hybrid modeling approaches. As rising temperatures accelerate permafrost thaw, changes in ALT can disrupt ecosystems, damage infrastructures, and enhance the release of stored soil carbon, highlighting the urgent need for improved predictive capabilities to inform adaptation strategies in the Arctic.

**Supplementary Information:**

The online version contains supplementary material available at 10.1038/s41598-025-26586-w.

## Introduction

Permafrost regions covers approximately 15% of the Northern Hemisphere, encompassing a total area of approximately 14 million km^2^^1^,. The active layer thickness (ALT), defined as the uppermost soil layer that thaws in the summer and freezes in winter, plays a vital role in permafrost ecosystems^2^. This layer serves as the primary zone for biogeochemical, hydrological and ecological processes^3^. Warmer temperatures across the northern hemisphere are projected to accelerate permafrost thaw and increase ALT in the continuous, discontinuous and sporadic permafrost regions^4,5^. Such changes in ALT pose significant risks to build infrastructure and may trigger the release of previously sequestered soil carbon, contributing to positive climate feedback mechanisms. However, substantial uncertainty remains regarding the extent of ALT changes, their primary drivers, and the broader implications for climate–carbon interactions^6^.

Understanding the permafrost active layer is critical, as it influences a wide range of processes essential to ecosystem functionality and climate feedback. While the active layer itself contains a relatively smaller portion carbon, it protects the permafrost, which holds a substantial amount of organic carbon, particularly within the top three meters of soil. As permafrost thaws under warming, previously frozen carbon becomes biologically available, leading to the potential release of greenhouse gases such as CO₂ and CH₄. Thus, monitoring the active layer can serve as an early indicator of permafrost degradation and other broader environmental implications. Additionally, changes in ALT significantly affect hydrological dynamics; for instance, increased ALT can lead to ground subsidence and destabilize infrastructure. These changes also influence surface hydrology, contributing to both drying and wetting patterns across permafrost regions, as shown in model intercomparison studies^7^.

To understand the response of the active layer and the near-surface permafrost to climate and environmental change, the Circumpolar Active Layer Monitoring (CALM) program was initiated in the 1990s. The CALM collects long-term observations of ALT, near-surface permafrost change, and associated climatic variables at over 200 sites across the circumpolar permafrost zone^8,9^. ALT is highly variable across space, influenced by a complex set of environmental factors, including vegetation cover, soil moisture and texture, thermal properties, organic layer thickness, snow cover, air temperature, topography, and human disturbance^10^. These seminal CALM datasets provide essential observational benchmarks for identifying spatial and temporal trends in permafrost thaw and serve as foundational inputs for calibrating and validating permafrost models. However, despite the availability of such detailed datasets, current Earth system models (ESMs) often fail to capture the fine-scale heterogeneity of ALT, contributing to large uncertainties in projections of permafrost carbon-climate feedbacks and ecosystem responses under future climate projections^11,12^.

Various models have been developed to estimate ALT, each offering unique advantages. The Geophysical Institute Permafrost Laboratory (GIPL) developed a physically based model that uses environmental factors like soil temperature, snow depth, and moisture to simulate permafrost dynamics but it is limited by its coarse resolution^13^. Whitcomb, et al.^14^ employed remote sensing and machine learning (ML) to upscale ALT measurements across Northern Alaska at a 30 m resolution for years 2014, 2015 and 2017. ML models excel in integrating diverse, high-resolution datasets to predict ALT by identifying complex, nonlinear relationships, making them highly adaptable and accurate but dependent on the quality of input data. Other permafrost modeling approaches include the Kudryavtsev model^15–17^, a process-based approach that simulates ALT through heat transfer and freeze-thaw dynamics, which provides a clear understanding of permafrost processes but typically at lower resolution^5^. Process-based models can be compared with ML models to gain better data-driven insights by integrating foundational physical principles for understanding ALT dynamics.

Random forest (RF) models have been demonstrated to be effective tools for predicting permafrost soil carbon and related soil properties^18,19^. Machine learning models including RF offer promising tools for capturing both linear and nonlinear relationships among environmental variables and observed ALT, enhancing the accuracy of ALT prediction. Traditionally, ALT has been estimated using classical approaches such as the Stefan model, which uses thermal properties (conductivity, latent heat, moisture, etc.)^20^^[,21^. However, this method heavily relies on temperature amplitude and mean annual temperature as key drivers, making it less reliable in years characterized by significant climatic anomalies. In addition to the physically based models, geographically weighted kriging has been used to predict ALT using the spatially distributed environmental covariates across permafrost regions^22,23^. Recent advancements have integrated remote sensing data to enhance ALT prediction, utilizing the vegetation characteristics derived from Landsat imagery, spectral indices, and biomass estimates^24–26^. In parallel, process-based ecosystem models such as the Ecosystem Model and the Terrestrial Ecosystem Model (TEM) have also been widely used to simulate ALT. These models estimate soil thermal regimes by explicitly modeling carbon and nitrogen cycling in relation to vegetation, soil properties, and climate conditions^27,28^. Additionally, large scale frame work including Earth System Models (ESMs)^29,30^, Land Surface Models (LSMs)^31^, surface frost index models^32^, and empirical approaches based on soil temperature and air temperature metrics^33,34^ have also been utilized to assess ALT. Comparing these diverse modeling approaches with emerging ML techniques offers a robust and integrative framework to improve our understanding of ALT dynamics under evolving climate conditions.

Recent work by Peng, et al.^5^ provided circumpolar-scale projections of ALT at 25-km resolution using a ML approach combined with Coupled Model Intercomparison Project Phase 6 (CMIP6) climate scenarios, offering valuable large-scale insights into permafrost dynamics. In this study, we focused on predicting ALT across Alaska under both baseline (2014) and future climatic conditions. Utilizing field observations and environmental factors datasets with a ML approach, we developed high-resolution (250-meter) ALT maps and projected decadal changes under two radiative forcing scenarios based on climate forcing data from the CMIP6. The primary objectives of this study were twofold: (a) to predict baseline ALT using the ML model and evaluate its performance in comparison to the Stefan approach, and (b) to assess future changes in ALT under both moderate and high radiative forcing scenarios as projected by CMIP6 models. These analyses aim to enhance our understanding of spatial and temporal variations in ALT and their implications for permafrost dynamics under a changing climate.

## Materials and methods

### Study area, active layer thickness observations and environmental datasets

We used ALT field observations from the Circumpolar Active Layer Monitoring (CALM) program across the state of Alaska (68 sites) for year 2014 (Supplementary Figure S1)^35^. To investigate the environmental drivers influencing ALT variability, we compiled a comprehensive set of environmental variables including topographic data that were derived from the U.S. Geological Survey’s Digital Elevation Model (DEM) at a 250-meter spatial resolution^36^. This DEM was used to calculate several topographic indices, including aspect, slope, flow accumulation, stream power index, sediment transport index, and topographic wetness index^37–39^. Historic climate data specifically, average annual temperature and precipitation at a 1-kilometer spatial resolution for long term climatology (1970–2000) were obtained from Fick and Hijmans^40^. Land use and land cover data, available at 250-meter resolution, were sourced from the European Space Agency^41^, and bedrock geology information was acquired from the global lithological map developed by Hartmann and Moosdorf^42^. All datasets were reprojected to a common Alaska Albers Equal Area (WGS84) grid and resampled to 250-m. Continuous variables were resampled using bilinear interpolation, while categorical variables were processed using the nearest-neighbor resampling method. All data preprocessing and spatial operations were performed using the raster package in the R programming language.

### Earth system model and emission scenarios

We evaluated the ALT projections using four ESMs from CMIP6. The models included in this study were: the Beijing Climate Center Climate System Model (BCC-CSM) with spatial resolution of 112-km, the Community Earth System Model developed by the National Center for Atmospheric Research (NCAR-CESM) with spatial resolution of ~ 125 km, the United Kingdom Earth System Model by the Met Office Hadley Centre (UKESM) with spatial resolution of ~ 125 km, and the Canadian Earth System Model (CanESM) with spatial resolution of ~ 285 km developed by the Canadian Centre for Climate Modelling and Analysis. These models were selected based on data availability and were resampled to a common spatial resolution of 250 m using bilinear interpolation. Temperature and precipitation projections from these models were downloaded and temporally aggregated into decadal raster for analysis. To represent baseline climate conditions, we used 2014 data, reflecting both the availability of spatially extensive CALM measurements and the final year of the CMIP6 historical simulation period. Future ALT projections were based on temperature and precipitation data derived from two CMIP6 Shared Socioeconomic Pathway–Radiative Forcing scenarios: SSP2-4.5, representing a stabilization scenario with moderate mitigation efforts (4.5 W/m² radiative forcing), and SSP5-8.5, representing a high-emission, fossil-fuel intensive pathway (8.5 W/m² radiative forcing). For future climate simulations, we calculated decadal averages of temperature and total annual precipitation by averaging 10 years of data for each decade, allowing for the representation of long-term climate trends in ALT projections.

### Stefan model for prediction of active layer thickness

We used the Stefan model to predict permafrost ALT across Alaska for both baseline and future periods, using climate forcing data from four CMIP6 ESMs^43,44^. The Stefan model estimates thaw depth based on a set of physical soil and climate parameters. Specifically, E factor in the model is a function of thermal conductivity of thawed soil (*k*_*t*_, W m^− 1^ °C^− 1^), the relationship between the air and surface freezing/thawing index *(n*_*t*_*)*, soil bulk density (*ρ*_*b*_, kg m^− 3^) and soil water content (*w*,* %)*. However, due to limited availability of temporally comprehensive soil property data across Alaska, the full parameterization of the Stefan model could not be implemented (Eq. i). To address this limitation, we adopted a simplified version of the Stefan model that assumes a temporally uniform E factor. In this approach, the soil factor (E) and the thawing index (*TI*), expressed in °C Day^− 1^ are used to estimate ALT. The baseline historic observed temperature dataset was used based on Muñoz-Sabater, et al. ^45^ ERA5-Land reanalysis datasets. The E factor was empirically derived by back-calculating it from observation-based ALT (cm) measurements and corresponding thawing index values derived from temperature datasets (Eq. iii). This simplification allows for scalable application of the Stefan model across broad spatial domains while maintaining reasonable accuracy in ALT estimation under different climate scenarios.

The spatial map of Alaska’s E factor was generated using kriging interpolation method, based on the baseline E-factor map and the climate-forcing data (temperature) from the four selected ESMs. The future ALT trajectory for the CMIP6-ESMs was derived using this map. To estimate future ALT trajectories, we employed a space-for-time substitution approach, wherein the thawing index (TI) was updated using future temperature projections from each CMIP6 ESM. Final ALT projections under future scenarios were derived as the ensemble mean of the outputs from all four ESMs, providing a robust estimate of potential ALT dynamics under changing climate conditions. The dataset was randomly divided into two subsets, with 70% used for model training and 30% for model testing to evaluate the model performance.

### Machine learning model for prediction of active layer thickness

**Fig. 1:**
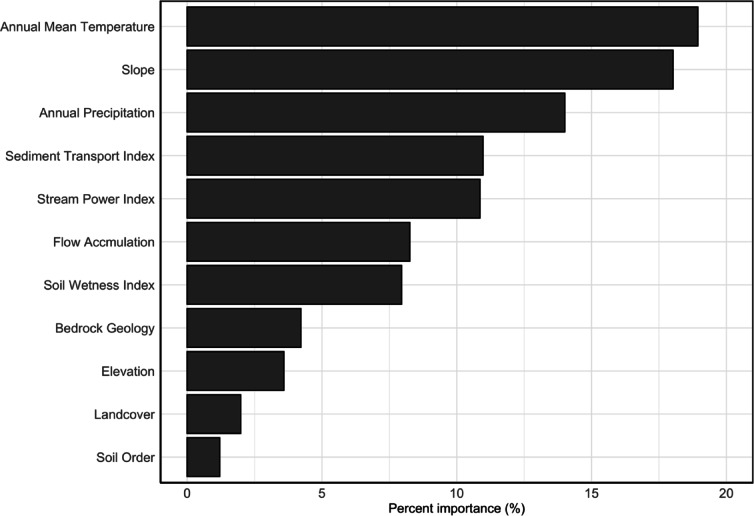
Variable importance of environmental predictors in the random forest model. The variable importance is scaled to represent percentage importance of each variable.

In this study, we employed a RF algorithm to predict ALT across Alaska at a spatial resolution of 250 m. Using observed ALT data from the CALM dataset (Supporting Information, Figure S1) and environmental variables (Fig. 1), we applied the RF model to predict ALT and rank the relative importance of climate, soil, topography and vegetation parameters in regulating its spatial distribution. Within the RF framework, each decision tree in the ensemble splits based on the most informative environmental covariates, which are randomly selected in subsets. Two critical model parameters: the number of variables randomly selected at each split (mtry) and the number of trees in the forest (ntree) were optimized using a 10-fold cross-validation. Based on this optimization, mtry was set to 5 and ntree to 500, minimizing the out-of-bag error and yielding the best model performance in terms of root mean square error (RMSE) and coefficient of determination (R²). Other parameters, such as nodesize and maxnodes, were kept at their default values after initial testing showed minimal impact on model accuracy compared to the optimization of mtry and ntree. Model tuning was conducted using the *caret* package in R.

1 $$E = \sqrt {\frac{{2{k_t}{n_t}}}{{pb{w^L}}}}$$

2 $$E = \frac{{ALT}}{{\sqrt {TI} }}$$

3 $$TI = \sum\nolimits_{(i = 1)}^{12} {{T_i},{T_i}> 0}$$

The dataset was randomly divided into two subsets, with 70% used for model training and 30% for model testing. Variable importance was assessed by measuring the change in predictive performance (expressed as changes in *R*^*2*^ and RMSE) when each predictor was randomly permuted, with results averaged across repeated permutations based on 10-fold cross-validation. Model validation was performed using the testing dataset, and spatial predictions were subsequently generated using gridded environmental variables across Alaska. For future ALT projections, we employed a space-for-time substitution approach, wherein future environmental variables (temperature and precipitation) derived from CMIP6 outputs were used in the RF model to estimate ALT changes under different climate scenarios. Model performance was evaluated using the coefficient of determination (*R²*) and the root-mean-square error (RMSE) between observed (O_i_) and predicted (P_i_) values.

4 $$\:RMSE=\sqrt{\frac{\sum\:_{i=1}^{n}{\left({P}_{i}-{O}_{i}\right)}^{2}}{n}}$$

5 $$\:{R}^{2}=1-\frac{\sum\:_{i=1}^{n}{\left({P}_{i}-{O}_{i}\right)}^{2}}{\sum\:_{i=1}^{n}{\left({O}_{i}-\stackrel{\prime }{O}\right)}^{2}}$$

Here, P_i_ and O_i_ are predicted and observed values, n is the number of observations, and $\:\overline{\text{O}}$ denotes the mean of observed values.

## Results and discussion

### Observed environmental controls of active layer thickness

The RF model achieved an *R*^*2*^ value of 0.84 for the training datasets and 0.24 for the testing dataset, with corresponding RMSE values of 14 cm and 22 cm, respectively. The average ALT across Alaska for the baseline year 2014 was 54 ± 23 cm, with a range from 25 to 168 cm based on the CALM dataset. Among the environmental predictors, mean annual temperature exerted the greatest influence on ALT distribution, accounting for 19% of the total variation based on the increase in residual sum of squares (Fig. 1). Slope gradient was similarly influential, explaining 18% of the variance in ALT. Annual precipitation accounted for approximately 10% of the variation in ALT distribution. Additionally, the sediment transport index and stream power index were identified as key contributors, explaining14% and 11% of the variation, respectively. The sediment transport index quantifies the potential for sediment transport as a function of upslope contributing area per unit contour width and local slope, and the stream power index represents the erosive power of flowing water. Other environmental factors including flow accumulation, soil wetness, bedrock geology, elevation, land cover, and soil order together explained the remaining variability in ALT. These results highlight the significance of both climatic and topographic controls in shaping the spatial patterns of ALT in permafrost regions. For the Stefan approach, the baseline soil factor (E) and the thawing index (*TI*) was used to calculate the ALT. The Stefan model achieved an *R*^*2*^ value of 0.53 for the training dataset and 0.54 for the testing dataset, with corresponding RMSE value of 17 cm and 18 cm, respectively.

Previous studies have consistently documented permafrost degradation as a function of climatic and topographic factors. For instance, Beltrami and Mareschal^46^ and Zhang, et al.47 showed a strong link between increasing surface air temperatures and the deepening of the active layer. Similarly, Sharkhuu^48^ observed that slope orientation significantly influences ALT dynamics in the Mongolian permafrost region, where south-facing slopes experienced greater warming and permafrost degradation due to increased solar radiation exposure compared to north-facing slopes. Therefore, topography also plays a critical role in permafrost vulnerability as it influences both hydrological processes and thermal dynamics^49^. For example, steeper slopes promote better drainage and reduced moisture retention, leading to deeper thaw depths^50^, while flatter surfaces often retain more moisture and facilitate shallower thawing due to the higher latent heat of fusion required to melt ice-rich soils^51^. Increased soil moisture enhances the soil’s thermal conductivity but dampens energy exchange with the atmosphere, further reducing thaw depth^52^. This is supported by Zhan, et al.^53^ who reported that elevation, slope angle, and soil moisture collectively shape the spatial heterogeneity of ALT across permafrost landscapes.

Subsurface warming associated with increasing winter air temperatures has been found to be a key contributor to long-term increases in ALT^54^. Schuh, et al.^55^ found that winter warming effects can penetrate up to 10 m below the surface, significantly altering the long-term ground thermal regime. On the Qinghai-Tibetan Plateau, Wu and Zhang^56^ found that ALT increased more rapidly in warmer versus cooler permafrost zones, indicating a non-linear response of permafrost to warming. Li, et al.^57^ further highlighted the dual role of precipitation in regulating ALT, as summer precipitation enhancing soil heat retention, leading to deeper thaw, while winter precipitation insulates the ground through increased snow cover, reducing heat loss and cooling the surface.

Landscape heterogeneity further influences ALT distribution. Shiklomanov, et al.^8^ analyzed spatial variations of ALT in Barrow, Alaska, and attributed observed differences to interactions among topography, vegetation cover, and soil moisture regimes. A broader circumpolar synthesis by Aalto, et al.^58^ and Aalto, et al.^59^ projected significant permafrost degradation under future warming, reaffirming temperature as the dominant control over ground thermal regimes.

Additionally, recent remote sensing and data assimilation approaches have enabled near-global ALT mapping, validating the role of terrain, vegetation, and climate in shaping ALT patterns^60^. Consistent with prior studies, our results on the sensitivity of different environmental factors indicate that permafrost degradation is a multifactorial process, driven by both climatic variables and local geomorphological characteristics. These findings highlight the importance of high-resolution, regionally calibrated models to accurately predict future permafrost dynamics.

### Estimation of ALT under current climate

**Fig. 2:**
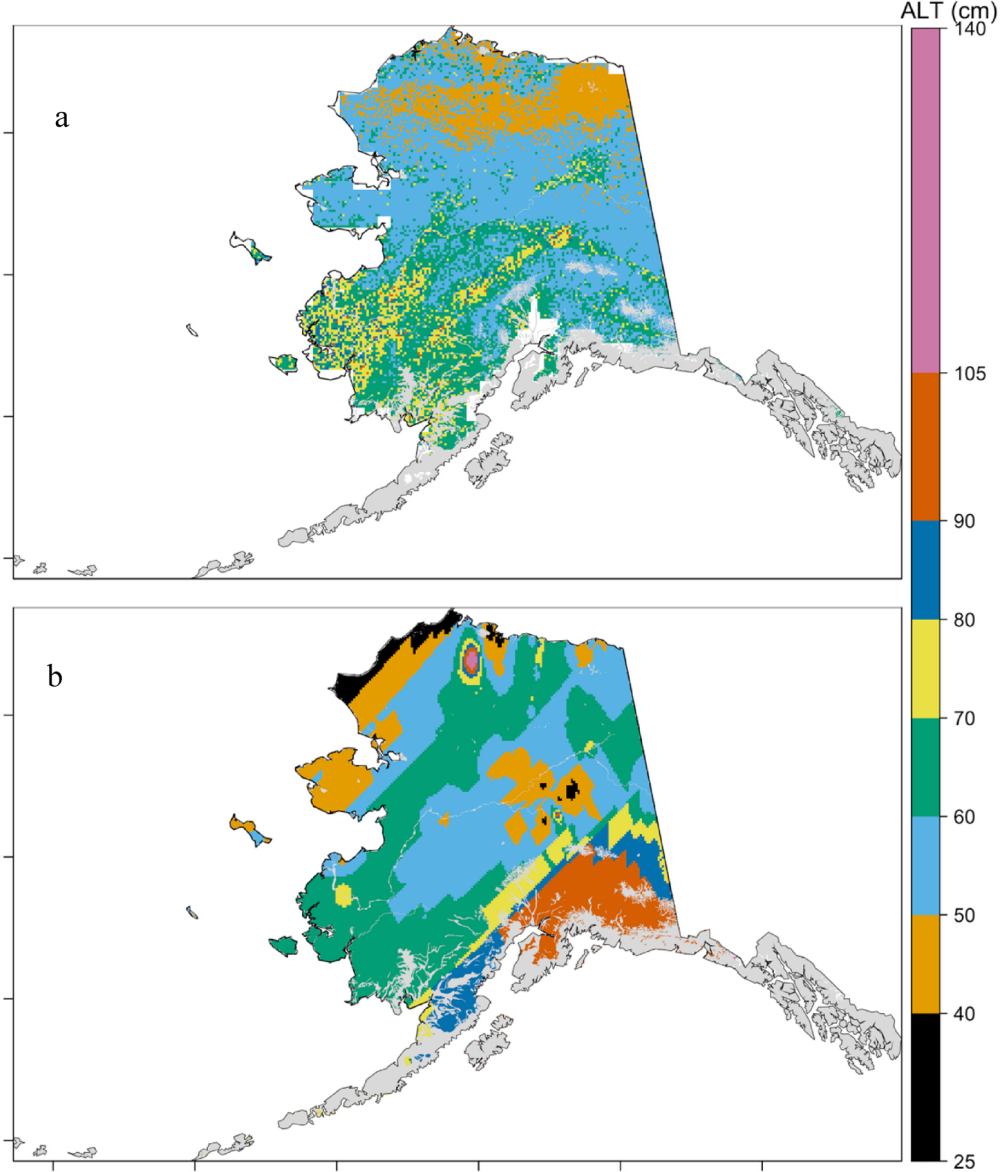
Comparison of the baseline active layer thickness (cm) predicted by random forest model (a) and the Stefan model (b). The grey region indicates areas where permafrost is absent. Maps were generated using R (version 4.3.2; https://www.r-project.org/).

The baseline (2014) predictions of ALT using both the RF-based estimation and the Stefan model-based estimation revealed notable differences in the estimated ranges and mean values (Fig. 2). The RF model predicted ALT values ranging from 36 to 102 cm, with a mean of 59 ± 8.8 cm (Fig. 2a). In contrast, the Stefan model estimated a wider range of ALT values, from 25 to 140 cm, with a higher mean of 65 ± 16 cm (Fig. 2b). Both models tended to overestimate baseline ALT relative to observed values, yet they consistently captured the expected latitudinal gradient in ALT, showing an increase in ALT from northern (high-latitude) to southern (lower-latitude) regions of Alaska. This spatial pattern is consistent with the distribution of mean annual air temperature, as previously reported by Jorgenson, et al.^61^, where warmer southern climates drive deeper seasonal thawing, while colder northern climates limit thaw depth. Comparisons with earlier studies further underscore these differences: the Geophysical Institute Permafrost Laboratory (GIPL) model of 1-km spatial resolution predicted a mean baseline ALT of 78 cm across Alaska^13^, Whitcomb, et al.^14^ while reported ALT values across northern Alaska ranging from 25 cm to 65 cm using a random forest approach, within the range of our RF-based predictions. Our comparison with the Arctic–Boreal Vulnerability Experiment (ABoVE) upscaled ALT dataset was restricted to the northern Alaska region overlapping with that product, corresponding primarily to the Arctic Coastal Plain and adjacent Boreal ecoregions. These differences across multiple studies highlight the influence of model structure, spatial resolution (30-m), and input data variability on ALT estimation.

A regional comparison between the RF and Stefan model predictions is summarized in Table 1. In high-latitude and mountainous regions such as the Brooks Range, Arctic Foothills, Ogilvie Mountains, and the Arctic Coastal Plain, the RF model estimated lower ALT values ranging from 49 to 53 cm, closely aligning with the Stefan model’s predictions of 49 to 59 cm. However, notable regional differences were observed in the areas with warmer climatic conditions. The RF model predicted the highest ALT values in the Subarctic Coastal Region and Bristol Bay–Nushagak Lowlands, whereas the Stefan model estimated maximum ALT depths in the Coastal Western Hemlock–Sitka Spruce Forests and the Pacific Coastal Mountains. These results suggest that while both models successfully capture broad spatial trends in ALT, the machine learning approach provides more spatially constrained estimates, likely due to its data-driven calibration using observed environmental controls. In contrast, the Stefan model, while conceptually sound, is more sensitive to assumptions and limitations in soil and thermal parameter availability, which can lead to regional overestimations in certain regions. These differences are reflected in the regional mean values and standard deviations of ALT predicted by the two models. The integration of both modeling frameworks to create physic-informed ML modeling framework offers complementary insights into ALT dynamics. However, future efforts should focus on model refinement through improved soil datasets, incorporation of snow insulation effects, and ensemble modeling strategies that account for both physical process knowledge and empirical observations.

**Table 1:** Comparison of baseline active layer thickness across ecoregions of Alaska using the machine learning based Estimation and Stefan approach.

Ecoregion	ML based ALT (cm)		Stefan based ALT (cm)	
	Mean	Standard Deviation	Mean	Standard Deviation
Ahklun and Kilbuck Mountains	68	6	63	2
Alaska Peninsula Mountains	67	2	83	7
Alaska Range	60	4	79	13
Arctic Coastal Plain	53	7	49	14
Arctic Foothills	52	5	55	15
Bristol Bay-Nushagak Lowlands	71	5	76	6
Brooks Range	49	3	59	6
Coastal Western Hemlock-Sitka Spruce Forests	68	2	98	4
Cook Inlet	69	5	88	8
Copper Plateau	64	6	96	1
Interior Bottomlands	69	8	58	7
Interior Forested Lowlands and Uplands	60	7	58	7
Interior Highlands	55	4	59	11
Ogilvie Mountains	52	2	59	3
Pacific Coastal Mountains	63	4	97	3
Seward Peninsula	57	4	50	4
Subarctic Coastal Plains	71	9	62	6
Wrangell Mountains	59	3	92	4
Yukon Flats	60	7	59	6

### Projection of ALT under future climate

**Fig. 3:**
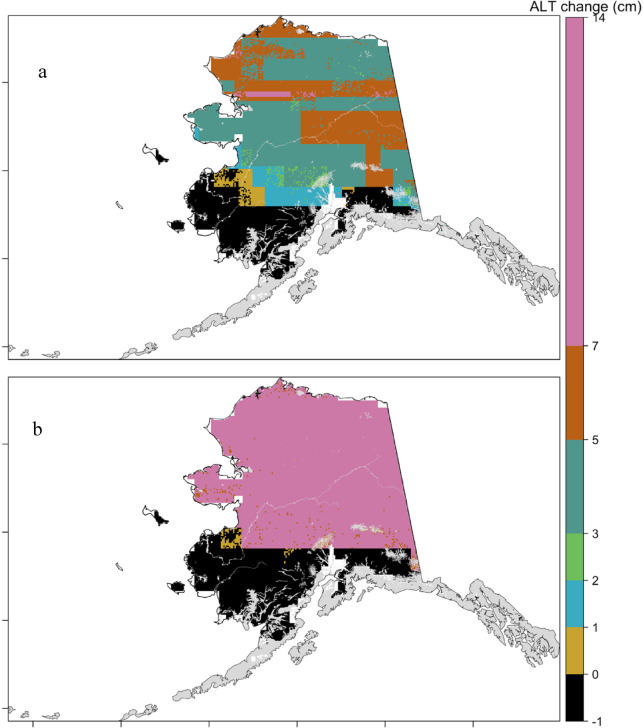
Comparison of the changes in the Active layer thickness (cm) between 2030 and 2100 based on random forest model (a) under SSP 245 emission scenarios and (b) under SSP 585 emission scenarios. The grey region indicates areas where permafrost is absent. Maps were generated using R (version 4.3.2; https://www.r-project.org/).

**Fig. 4:**
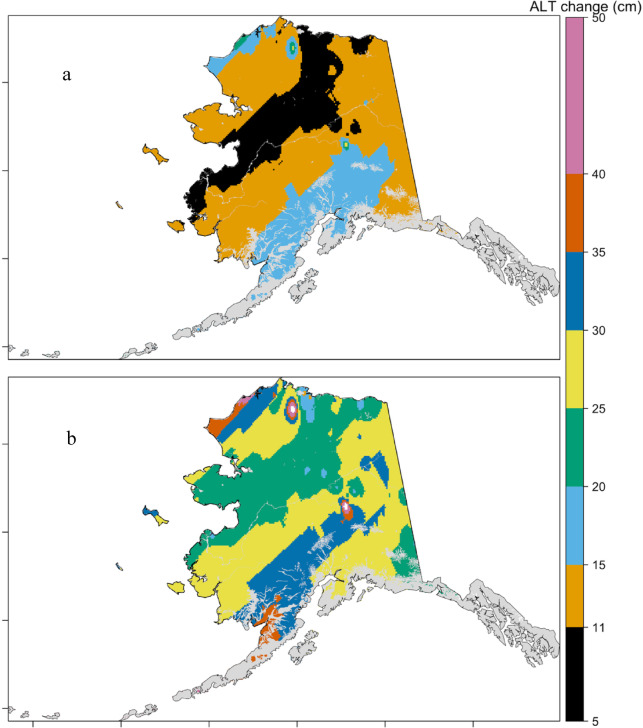
Comparison of the changes in the Active layer thickness changes (cm) between 2030 and 2100 based on Stefan model (a) under SSP 245 emission scenarios and (b) under SSP 585 emission scenarios. The grey region indicates areas where permafrost is absent. Maps were generated using R (version 4.3.2; https://www.r-project.org/).

**Fig. 5:**
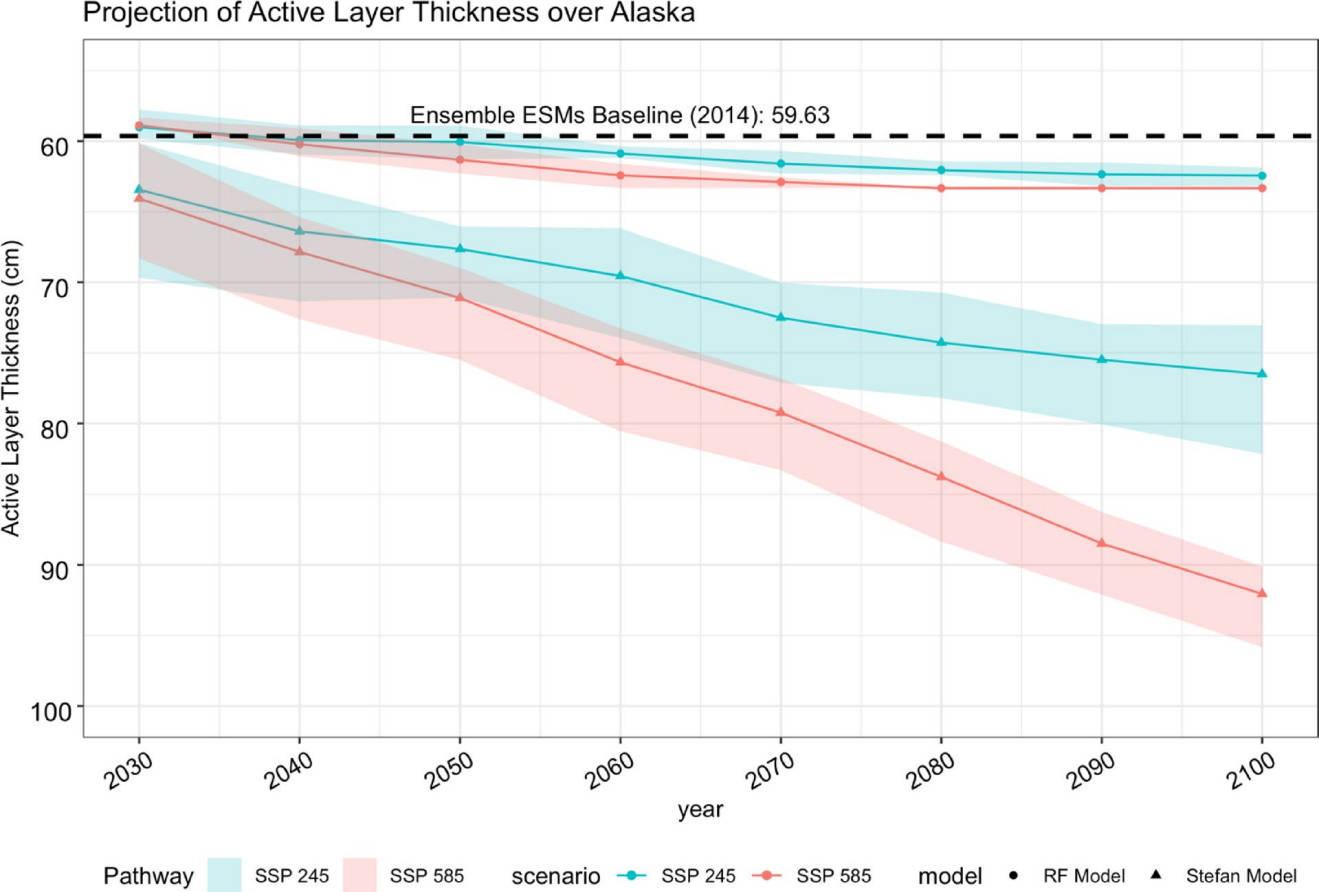
Comparison of decadal changes in active layer thickness between 2030 and 2100 for both the Random Forest model and the Stefan model under two emission scenarios (SSP2-4.5 and SSP5-8.5). The solid lines represent the median values, while the shaded areas indicate the interquartile range (25th to 75th percentiles).

Projected changes in ALT throughout the 21 st century indicate a consistent increase in response to future changes in air temperature, as estimated by both the Stefan model and the ML approach (Supplementary Figs. 2 and 3). The ML-based projections suggest a mean ALT increase of 3.3 ± 2.2 cm under the SSP2-4.5 and 5.9 ± 4 cm under the SSP5-8.5 scenario between 2030 and 2100 (Fig. 3). In contrast, the Stefan model projects substantially higher increases, with median values of 13 ± 2.6 cm under SSP2-4.5 and 28 ± 4.4 cm under SSP5-8.5 (Fig. 4), displaying a banded pattern, with an interior arc of lower thaw-depth change and higher changes along coastal regions, consistent with regional temperature gradients. The ML model also showed slight (up to 1 cm) decreases in ALT, in certain isolated and sporadic permafrost regions. Figure 5 presents the ensemble trajectories of ALT derived from both the Stefan model and the random forest model across future decades, relative to the 2014 baseline. The figure illustrates both the mean values and interquartile ranges. The projected ALT increase in this study is smaller than reported in previous process-based modeling studies. This difference likely reflects the empirical nature of the Random Forest model, which is constrained by historical relationships and may underrepresent nonlinear feedback. Differences in spatial resolution, temporal scope, and input forcing may also contribute, suggesting that our results likely represent conservative estimates of future ALT change. Both models consistently project the most substantial ALT increases at higher latitudes, while predicting relatively minor changes at lower latitudes. The conservative ALT increases projected by the ML model are likely influenced by its limited extrapolation capacity beyond the historical training data ranges^62^. In contrast, the Stefan model, responds linearly to projected temperature increases due to its physically based formulation, resulting in higher ALT estimates. These findings are consistent with previous studies. For instance, ^13^ projected mean ALT values of 133 cm by 2050 and 240 cm by 2100 in Alaska under the CMIP3 A1B scenario using the GIPL model. Peng et al. (2018) reported decadal increases of 2.56 cm and 6.51 cm under RCP4.5 and RCP8.5, respectively, across the Northern Hemisphere using the Stefan model. Yi, et al. ^11^ observed regionally variable ALT increases, with slower rates (~ 0.32 cm/year) in northern Alaska and faster increases (> 3 cm/year) in interior and southern Alaska. More recently, Peng, et al. ^5^ estimated that under RCP 8.5, near-surface permafrost extent may decline by 14% at 3.5 m depth and by 1.3% at 6.0 m, indicating widespread permafrost degradation by century’s end.

Our study has certain limitations and underlying assumptions. First, the random forest model was developed using a relatively small set of observational data obtained from the Circumpolar CALM network. Due to the limited size of the dataset, we performed parameter tuning using the full dataset, which may introduce a degree of information leakage. The limited number of ground-truth observations may reduce the generalizability and accuracy of the model predictions across spatial scales. Additionally, the Stefan model used in this study relies mainly on-air temperature as its primary input variable. While this approach provides a simplified estimation of ALT, E factor aggregates effects of soil properties, organic layer thickness, snow insulation, and surface energy balance; treating it as constant in time can misrepresent evolving snow/vegetation regimes. As a result, the Stefan model’s projections may not fully capture the complex interactions that influence permafrost dynamics. To enhance the robustness of future projections, research should explore hybrid modeling approaches that integrate machine learning with physically based models such as the Stefan model. Additionally, this study used CMIP6 data without downscaling, which may limit representation of local climate variability influencing ALT projections. Projected increases in ALT pose significant risks to infrastructure and may accelerate soil carbon release, contributing to positive climate feedback.

## Conclusions

This study utilized both a ML model and a process-based Stefan model to predict ALT and investigate its key environmental controls across Alaska. The random forest model outperformed the Stefan approach in predicting ALT, achieving higher accuracy for the training dataset (R² = 0.84 vs. 0.53) but lower generalizability on the testing dataset (R² = 0.24 vs. 0.54), with respective RMSEs of 14–22 cm and 17–18 cm. Mean annual temperature and slope angle were identified as the dominant drivers, accounting for 19% and 18% of the total variation in ALT, respectively in ML model. Other terrain-based factors such as the sediment transport index and stream power index also contributed significantly. Model comparisons revealed systematic differences in performance: the Random Forest model tended to underestimate ALT, whereas the Stefan model relying solely on thermal dynamics generally overestimated it due to its simplified assumptions. Nonetheless, both models captured a consistent spatial trend of increasing ALT from higher to lower latitudes. Future projections under two shared socioeconomic pathways (SSP 2–4.5.5 and SSP 5–8.5.5) indicated a consistent increase in ALT across Alaska. The ML model predicted mean ALT increases of 3.3 ± 2.2 cm and 5.9 ± 4 cm under SSP 2–4.5.5 and SSP 5–8.5.5, respectively, while the Stefan model projected substantially larger increases of 13 ± 2.6 cm and 28 ± 4.4 cm for the same scenarios. The largest changes in ALT were found in higher latitudes, indicating strong regional variability in permafrost sensitivity to environmental change. These results highlight the importance of integrating diverse environmental variables into ALT models to improve predictive accuracy and reduce uncertainty. Improved modeling of permafrost dynamics is essential for anticipating the cascading impacts of thawing permafrost on Arctic ecosystems, infrastructure integrity, and global carbon-climate feedback. Future research should prioritize the incorporation of high-resolution observational data and explore the broader ecological and climatological implications of permafrost degradation under accelerating climate change.

## Supplementary Information

Below is the link to the electronic supplementary material.

Supplementary material 1 (DOCX 7016.8 kb)

## Data Availability

Data is provided within the manuscript & supplementary information files.
