# Supplementary material for: Machine learning and process-based modeling of spatiotemporal changes in active layer thickness across Alaska
Source: Sci Rep. 2025 Nov 27;15:42420. doi: 10.1038/s41598-025-26586-w (PMC12660986; doi:10.1038/s41598-025-26586-w)
Supplement: Supplementary file 1 — Supplementary material 1 (DOCX 7016.8 kb) [file 41598_2025_26586_MOESM1_ESM.docx]

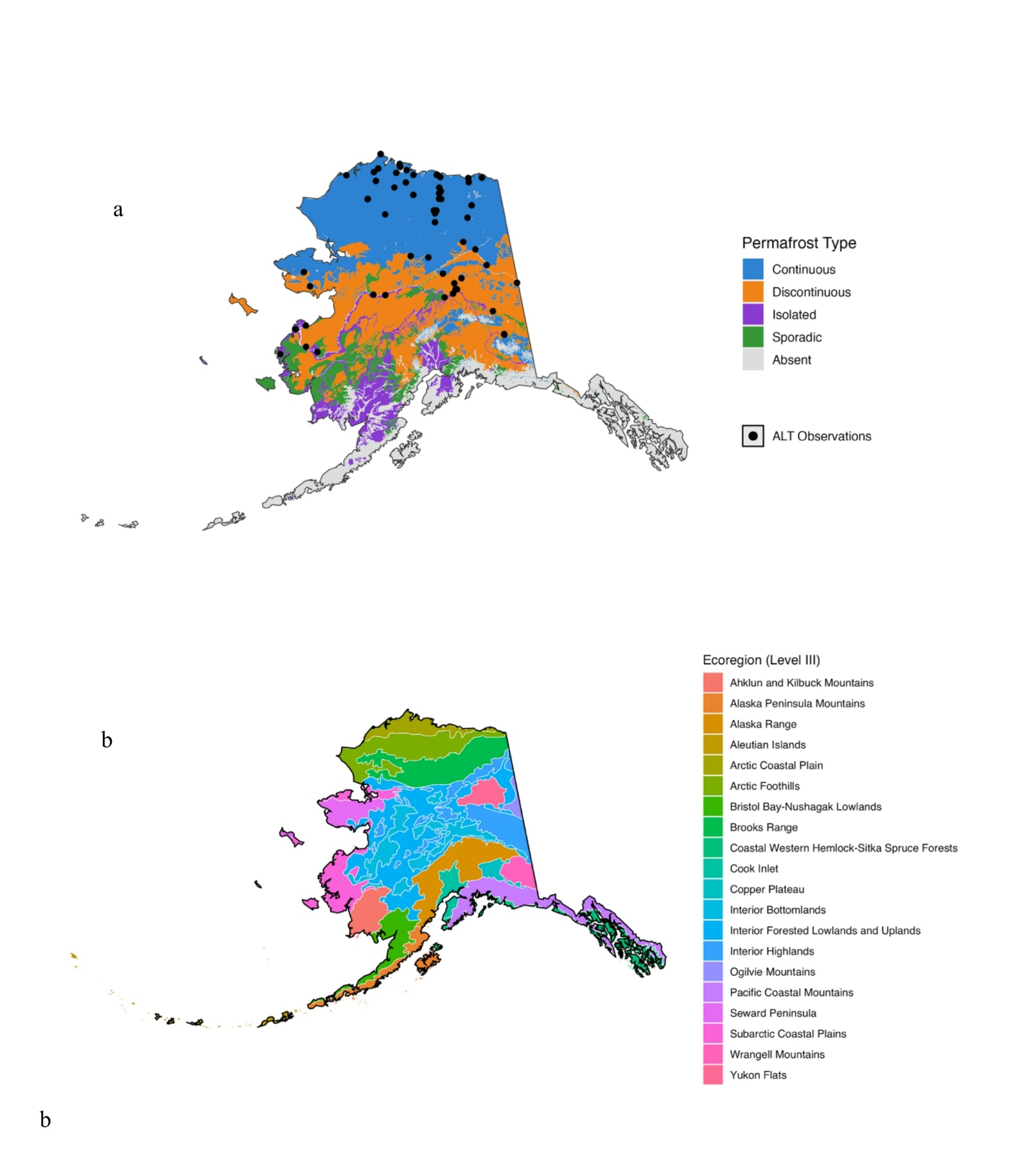



**Supplement Figure S1:** (a) Permafrost zones and locations of Circumpolar Active Layer Monitoring (CALM) sites across Alaska; (b) Level III ecoregions of Alaska. Maps were generated using R (version 4.3.2; https://www.r-project.org/) using Active Layer Thickness, Version 2 dataset (NSIDC; https://nsidc.org/data/ggd318/versions/2).


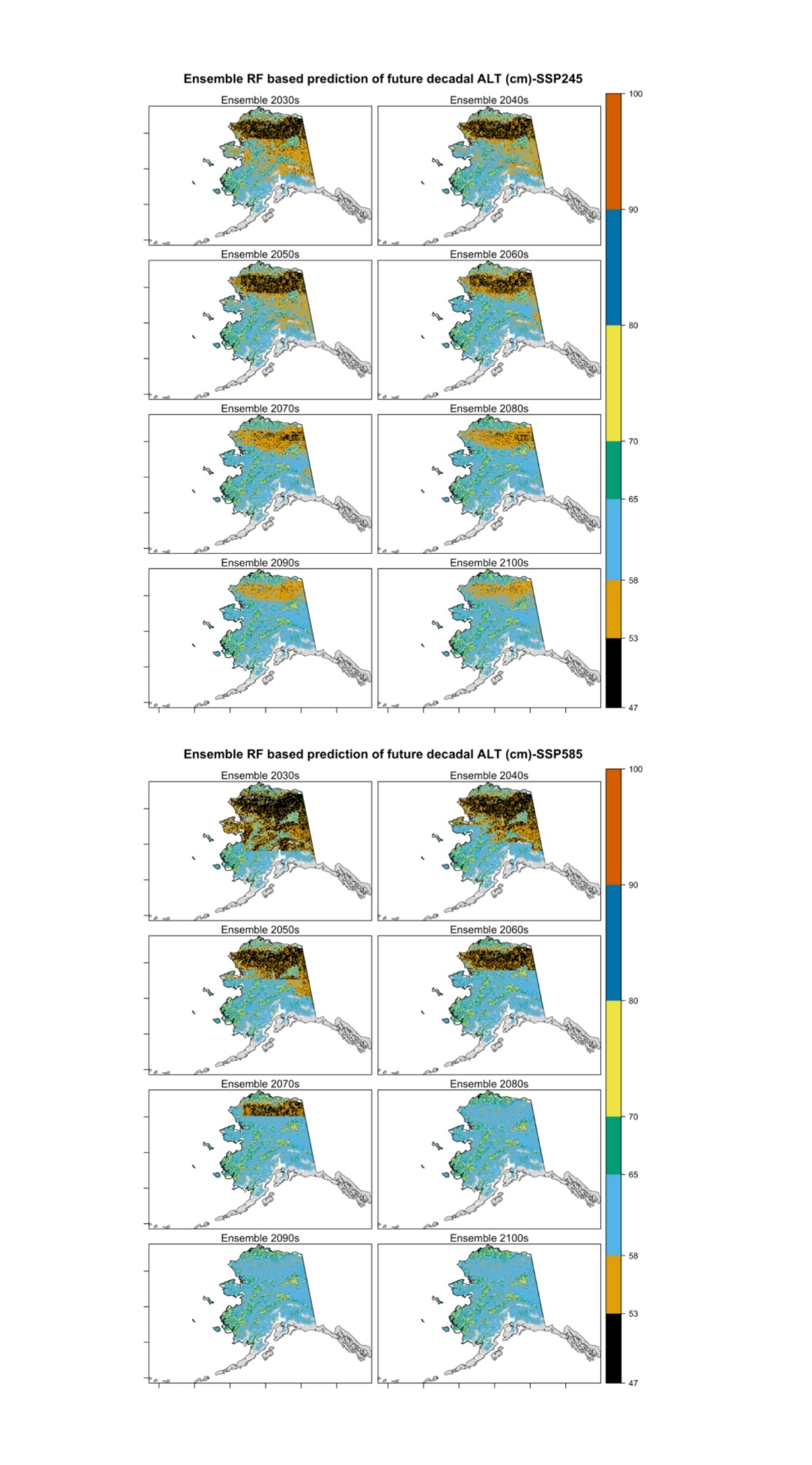


**Supplement Figure S2:** Projected decadal changes on active layer thickness based on random forest model for two emission scenarios (SSP 245 and SSP 585). The grey region indicates areas where permafrost is absent. Maps were generated using R (version 4.3.2; https://www.r-project.org/).


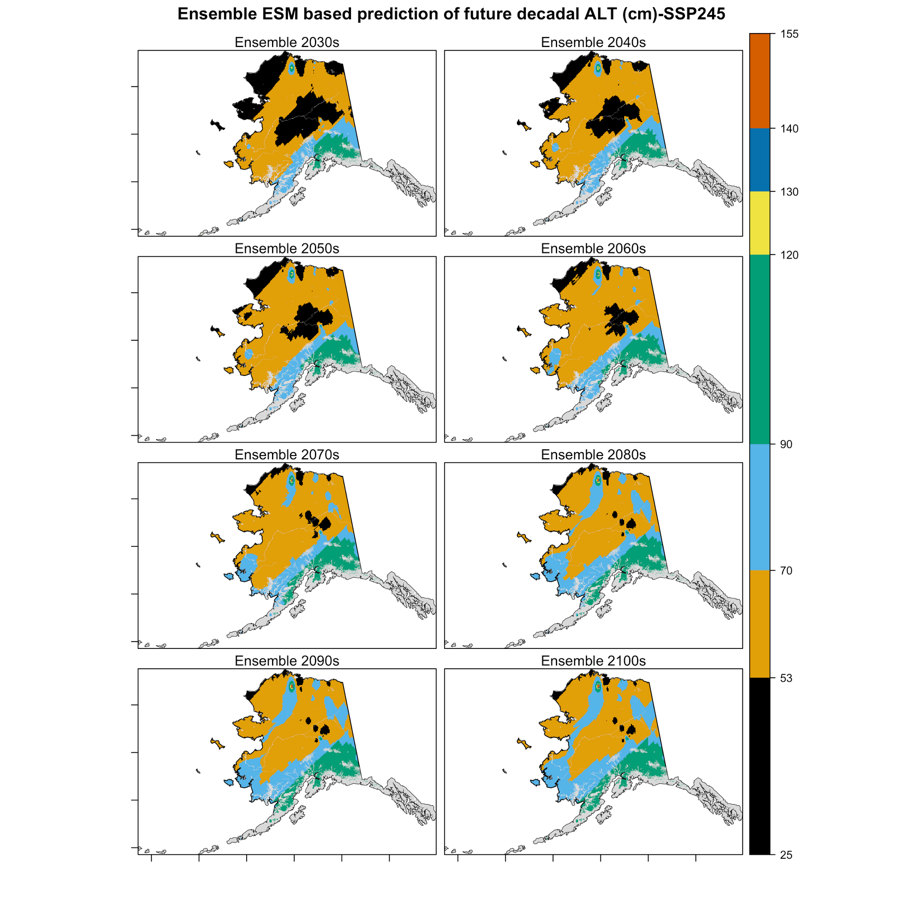


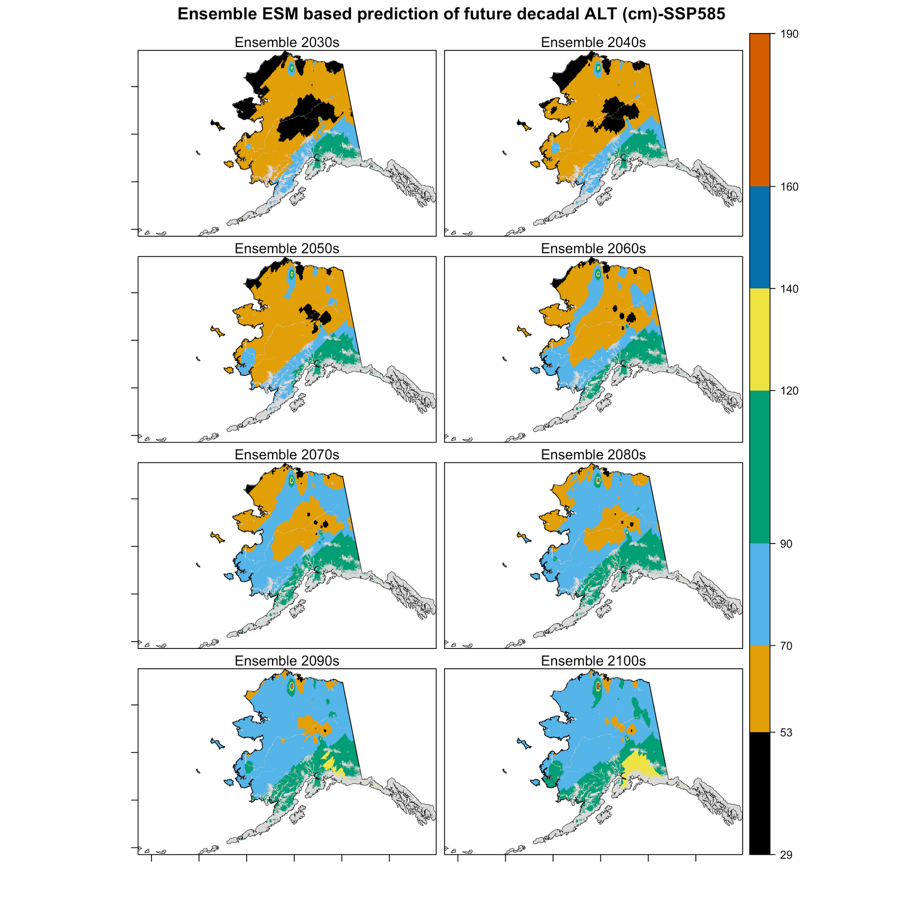


**Supplement Figure S3:** Projected decadal changes on active layer thickness based on Stefan model for two emission scenarios (SSP 245 and SSP 585). The grey region indicates areas where permafrost is absent. Maps were generated using R (version 4.3.2; https://www.r-project.org/).
